# The rehabilitation in the management of Transthyretin Familial Amyloid Polyneuropathy

**DOI:** 10.1186/1750-1172-10-S1-P66

**Published:** 2015-11-02

**Authors:** Agnès Morier, Alyssa De Sousa, Colombe Lemoine, Cecile Cauquil, Marie Théaudin, David Adams, Hervé Chanut

**Affiliations:** 1 Centre de Références Neuropathies Rares (NNERF), service de Neurologie Adulte, Univ Paris Sud, CHU Bicêtre, 78 rue du Général Leclerc 94275 Le Kremlin Bicêtre, Paris, France; 2 Equipe de rééducation du Centre de Références Neuropathies Rares (NNERF), CHU Bicêtre, 78 rue du Général Leclerc 94275 Le Kremlin Bicêtre, Paris, France

## Introduction

The rehabilitation is part of a drug free therapy management of peripheral polyneuropathy. The Transthyretin Familial Amyloid Polyneuropathy this illness entails deficiencies that do impact on the day to day physical comfort and everyday life of patients. They have motor function and sensory consequences.

## Methods

To be able to offer a well-adapted rehabilitation program, the rehabilitation therapists have put into place assessments in order to estimate the different clinical manifestations described by the patients. The pain, the paresthesia and strength deficiency will be evaluated in this way, and their evolution followed up, thanks to comparative test. We have been looking for tools to be able to evaluate the clinical manifestations and their evolution.

## Results

For that purpose, we have chosen comparative tests that allow to measure quantitative and qualitative results. The pain and the tiredness are evaluated with visual analogue scales, the strength with muscular testing and dynamometer, the functional aspect is tested with an evaluation of: the standing upright; the handicap situation and the 6 minute walking test. The assessments' have improved in recent years with more precise tools.

## Conclusion

It is essential to stay as close as possible to the felt effects of the patients to deal with unbiased variations and put into place an adapted rehabilitation program.

